# Coping Strategies and Sense of Care Among Parents of Children Affected by Sturge–Weber Syndrome: A Cross-Sectional Study

**DOI:** 10.3390/nursrep16020066

**Published:** 2026-02-14

**Authors:** Hernández de Benito Alberto, Buceta Toro María Isabel, Sanz Guijo María, Serrano Gallardo María Pilar

**Affiliations:** 1 Nursing Department, Faculty of Nursing and Phisiotherapy Salus Infirmorum, Pontifical University of Salamanca, 28015 Madrid, Spain; ahernandezde@upsa.es (H.d.B.A.); msanzgu@upsa.es (S.G.M.); 2 Nursing Department, Faculty of Medicine, Autonomous University of Madrid, 28029 Madrid, Spain; pilar.serrano@uam.es; 3 Puerta de Hierro-Segovia de Arana Health Research Institute (IDIPHISA), 28222 Madrid, Spain; 4 Interuniversity Institute “Advanced Research on Evaluation of Science and the University” (INAECU), 28029 Madrid, Spain

**Keywords:** rare diseases, Sturge–Weber syndrome, caregivers, mothers, parents, meaning in caregiving, coping skills

## Abstract

**Background/Objectives:** The diagnosis of a rare disease such as Sturge–Weber syndrome (SWS) has a profound emotional impact on parents, who must adapt to an unexpected and complex caregiving role. This study aimed to analyse the sense of caregiving among parents of children with SWS and to identify the coping strategies they adopt. **Methods:** A cross-sectional descriptive study was conducted with 28 parents of children with SWS in Spain. Data were collected using the Brief COPE inventory and the Finding Meaning Through Caregiving Scale (FMTCS). Descriptive statistics and non-parametric bivariate analyses were performed. **Results:** Acceptance (mean = 5.14; SD = 0.85) and active coping (mean = 5.07; SD = 1.12) were the most frequently used coping strategies. Higher provisional meaning in caregiving was positively associated with active coping (ρ = 0.423; *p* = 0.025), acceptance (ρ = 0.562; *p* = 0.002), and humor (ρ = 0.557; *p* = 0.002). As children aged, parents reported a greater sense of caregiving meaning (ρ = 0.294; *p* = 0.049). **Conclusions:** Parents of children with SWS tend to adopt active and adaptive coping strategies over time, finding increasing meaning in their caregiving role. These findings highlight the importance of nursing-led interventions aimed at supporting parental coping, meaning-making, and emotional well-being in families affected by rare diseases.

## 1. Introduction

Rare diseases are defined as “those that, posing a risk of death or chronic disability, have a prevalence of fewer than 5 cases per 10,000 inhabitants,” according to the European Union’s expert committee [1]. These figures indicate that over 300 million people worldwide are affected by at least one of the 6000 known rare diseases, with pediatric onset in most cases and a genetic etiology in approximately 70% of instances [2].

Sturge–Weber syndrome (SWS) is a congenital, sporadic neurovascular disorder most often caused by a mutation in the GNAQ gene on chromosome 9q21 [3]. In Europe, the prevalence is estimated at 1 in 20,000–50,000 live births [4]. The main vascular malformations associated with this syndrome include a facial capillary malformation (port-wine stain on the upper part of the face), glaucoma, and a leptomeningeal malformation in the brain, which is the primary cause of seizures in the first months of life [5].

Due to the severity and extent of these malformations, each individual with SWS will present a different degree of impairment. Possible complications include hemiparesis or acquired vision loss [6,7,8,9], cerebral atrophy and calcifications [10], stroke events [11], intellectual disabilities [12], odontological manifestations [13] and autism spectrum disorders [14,15].

SWS is a multisystemic disorder requiring affected individuals to receive care from various specialists. A multidisciplinary team is essential, comprising experts specializing in endocrinology, psychiatry, dermatology, and ophthalmology, as well as providing rehabilitation services for mobility, motor skills, and speech therapy. In addition, many patients with Sturge–Weber syndrome experience drug-resistant epilepsy, which may require surgical evaluation and intervention. Therefore, neurosurgeons and neurologists play a crucial role within the multidisciplinary team, working alongside other specialists to optimize seizure control and neurological outcomes [16,17,18,19,20,21].

This disease profoundly impacts both patients and their families at biological, psychological or emotional levels. The diversity and variability of symptoms and disease progression lead to a highly individualized clinical presentation, with each case manifesting differently [22].

The diagnosis of a rare disease has a major emotional impact on the entire family, particularly on the mother and father, as it unexpectedly disrupts the family biography and demands a profound restructuring of lifestyle, expectations, and parental roles, thereby transforming the emotional, social, and functional dynamics of family life. The required adaptations may include implementing medical or surgical treatments, dietary changes, work adjustments, and a reorganization of daily routines [23,24].

Beyond its clinical severity, SWS also generates complex psychosocial challenges that extend to family functioning and parental adjustment. In addition to medical and neurological difficulties, children with Sturge–Weber syndrome frequently present cognitive, emotional, and social impairments that significantly affect their participation in daily life. Neurodevelopmental deficits, epilepsy-related limitations, autism spectrum features, and psychiatric symptoms are associated with reduced social competence and peer interaction [12,14,15]. In this context, parents assume a central role not only in managing complex healthcare needs but also in promoting social skills, facilitating inclusion, and mediating social relationships. These extended caregiving responsibilities, combined with visible physical manifestations and recurrent hospitalizations, increase parental burden and contribute to elevated stress and emotional vulnerability [25,26,27].

Having a child with a rare disease triggers a unique coping process, distinct from coping with one’s own illness, as it evokes intense feelings of vulnerability and loss of control—amplified by the scarcity of available information, limited access to specialists, and the lack of specific treatments—resulting in a pervasive sense of helplessness and uncertainty. The diagnostic delay, common in rare diseases, often intensifies the emotional burden experienced by parents, who must navigate long periods of uncertainty before obtaining a definitive answer [25].

In this context, parents may experience overwhelming emotions such as guilt, fear of their child’s death, and stress arising from the unpredictability of the clinical course and the perceived loss of the “ideal child” imagined before the diagnosis. Feelings of insecurity, sadness, frustration, anger, and hopelessness are also common, often coexisting with denial and other defense mechanisms that aim to mitigate the immediate emotional shock [25].

As the process evolves, more adaptive coping strategies tend to emerge, oriented toward seeking information and social support, although these are frequently accompanied by physical and mental exhaustion. Coping strategies become essential and are closely related to the care provided to the child as well as to the overall quality of life of the family unit [26,27]. Parental adjustment depends not only on available resources but also on cognitive appraisals and meaning-making processes that influence resilience and well-being [23,24]. Within this framework, the concept of “sense of caregiving” reflects parents’ efforts to integrate emotional burden, competence, and purpose into a coherent understanding of their role.

Although previous studies have explored caregiver stress, burden, and coping in families affected by rare diseases, evidence focusing on how coping strategies interact with caregiving meaning in parents of children with Sturge–Weber syndrome remains scarce. This gap limits the development of targeted nursing and psychosocial interventions tailored to the specific needs of these families.

The aim of this study was to analyze the sense of caregiving among parents of children affected by Sturge–Weber syndrome. Specifically, the study sought to identify the coping strategies adopted by parents and to explore the associations between coping strategies, sense of caregiving, and selected sociodemographic variables.

## 2. Materials and Methods

Given the low prevalence of Sturge–Weber syndrome and the limited number of families affected in Spain, this study was designed as an exploratory analysis. The final sample size reflects the accessible population through the national patient association and is consistent with previous research conducted in rare disease contexts. Due to the limited sample size, the study was not powered to perform multivariable regression analyses; therefore, bivariate non-parametric tests were used to explore associations between coping strategies and sense of caregiving. The findings should be interpreted as hypothesis-generating rather than confirmatory.

### 2.1. Design

This is a cross-sectional descriptive study conducted with parents of children affected by Sturge–Weber syndrome in Spain.

### 2.2. Participants and Settings

The study aimed to reach the entire population of parents with children affected by Sturge–Weber syndrome by recruiting participants through the Asociación Española de Sturge Weber (AESSW). This approach was chosen because most affected individuals in Spain are concentrated within patient associations, where parents seek others experiencing similar situations to exchange relevant disease-related information and provide mutual emotional support [28].

### 2.3. Data Collection

Data were collected using an online, fully voluntary, and anonymous questionnaire, which was disseminated through AESSW. Once initial contact was established via email with the principal investigator, the association distributed the questionnaire to its members through electronic communication channels.

The sociodemographic variables included in the questionnaire were: parental role (mother or father), age, family type based on whether both parents cohabited (as described by Irueste et al.: nuclear, single-parent, separated parents, or blended family), age of the affected child, number of children, birth order of the affected child, education level (compulsory education, secondary education, vocational training, or university studies), and employment status (subsidy for child care [CUME], unemployment, part-time employment, or full-time employment). The latter two categories were created based on the Spanish Labour Force Survey from the National Institute of Statistics [29].

Additionally, two validated instruments were used to assess coping strategies and the sense of meaning in caregiving: the Brief COPE scale [30,31] and the Finding Meaning Through Caregiving Scale (FMTCS) [32,33], both adapted for the Spanish population.

The Brief COPE was selected because it allows the assessment of a wide range of cognitive and behavioral coping strategies commonly used in response to stressful life events, including chronic illness caregiving. The FMTCS was chosen due to its specific focus on meaning-making processes in caregiving contexts, capturing both positive and negative dimensions of the caregiving experience. The combined use of these instruments enables a comprehensive analysis of coping strategies and sense of caregiving among parents facing long-term and complex care demands.

### 2.4. Brief COPE

The Brief COPE questionnaire was used to measure the coping strategies parents employed in response to the diagnosis and progression of their child’s disease. It consists of 28 items subdivided into 14 different coping styles, each rated on a 4-point ordinal Likert scale ranging from 0 (“I never do this”) to 3 (“I always do this”), with intermediate scores. Each coping style has a minimum score of 0 and a maximum of 6, as defined by Carver [30] and translated into Spanish by Morán Astorga et al. [31]. The 14 resulting subscales are: active coping, planning, use of instrumental support, use of emotional support, self-distraction, venting, behavioral disengagement, positive reframing, denial, acceptance, religion, substance abuse, humor and self-blame.

### 2.5. Finding Meaning Through Caregiving Scale (FMTCS)

This scale aims to identify both positive and negative feelings and how each parent copes with caregiving, seeking meaning in their own existence through caregiving. The Spanish adaptation by Fernández Capo and Gual García [33] was used, based on the original version by Farran et al. [32].

The FMTCS is a self-administered Likert-type scale consisting of 43 items, each rated from 1 to 5 according to agreement or disagreement. The scale is structured into four subscales: provisional meaning, loss/powerlessness, ultimate meaning and total meaning.

### 2.6. Data Analysis

All statistical analyses were conducted using IBM SPSS Statistics version 22. Prior to inferential analyses, data were screened for missing values, outliers, and distributional assumptions. Normality of each variable was assessed using Shapiro–Wilk tests and inspection of Q–Q plots. Because several variables did not meet normality assumptions, non-parametric tests were applied. No data transformations were necessary.

Outliers were examined using standardized residuals (|z| > 3.29) and Cook’s D (>4/n); analyses were replicated, including and excluding these cases, yielding consistent results. Missing data were below 5% and were handled by pairwise deletion.

Descriptive statistics were used to summarize the variables: measures of central tendency (mean or median) and dispersion (standard deviation [SD] or interquartile range [IQR]) for quantitative variables, and absolute frequencies and percentages for qualitative variables.

For the bivariate analyses, Mann–Whitney U and Kruskal–Wallis H tests were used to explore the influence of sociodemographic characteristics on coping styles and sense of caregiving. Associations between sense of caregiving and coping styles were examined using Spearman’s rank correlation coefficient (ρ) with 95% confidence intervals.

The significance threshold was set at *p* = 0.05 (two-tailed). Given the exploratory nature and small sample size typical of rare-disease research, no correction for multiple comparisons was applied; exact *p*-values are reported to allow interpretation of potential type I error inflation.

Because of the limited sample (n = 28), multivariable regression analyses were not conducted. Instead, bivariate non-parametric tests were used to explore associations between coping strategies and sense of care.

Internal consistency analyses were not recalculated for the present sample due to its limited size. Reliability values reported in the original validation studies of the Spanish versions of the Brief COPE and the FMTCS were therefore considered.

## 3. Results

The sample consisted mainly of middle-aged parents, most of whom lived in nuclear families and were employed full-time. Detailed sociodemographic characteristics are presented in Table 1.

A total of 82.1% of participants identified as belonging to a nuclear family, defined as one in which both parents cohabit in the family home. Regarding employment status, the majority of participants (n = 17) were employed full-time. Nearly half of the participants had a university-level education, with similar proportions distributed across other levels of education. The maximum number of children per family was three (reported by nine participants, 32.1%). The birth order of the child diagnosed with Sturge–Weber Syndrome within their siblings was second in 50% of cases (Table 1).

### 3.1. Brief COPE

The most frequently used coping strategies among parents of children with Sturge–Weber syndrome, based on their mean scores, were acceptance, with a mean of 5.14 (SD: 0.848), and active coping, with a mean of 5.07 (SD: 1.120). These were followed by positive reframing, which also had a mean score above 4 points.

In contrast, denial, behavioral disengagement, and substance use were rarely employed by participants, with mean scores below 1 point (Table 2).

Older parents tended to use coping strategies such as humor and venting. In addition, the age of the child was positively associated with acceptance, instrumental and emotional support, positive reframing, humor, and venting (Table 3).

#### Associations

The bivariate analysis of the scale revealed differences in coping style means between fathers and mothers, although these differences were not statistically significant. However, regarding family type, statistically significant differences were observed in self-distraction (5.0 vs. 3.0 and 1.8; *p* = 0.035) and substance use (0.5 vs. 0 and 0; *p* = 0.002), with higher means among separated parents compared to other family types. Additionally, parents who remained together showed lower levels of denial compared to those facing the disease individually (median scores: 0.2 vs. 2.0 and 1.5; *p* = 0.055). Although this difference did not reach statistical significance, it suggests a non-significant trend toward higher denial levels among parents facing the disease individually.

Parental employment status was also found to influence coping strategies, particularly positive reframing (*p* = 0.005). Parents employed full-time (4.9; SD: 1.0) were more likely to engage in this coping mechanism, focusing on growth and identifying positive aspects in their child’s condition. Additionally, participants working part-time exhibited higher substance use (0.3 vs. 0; *p* = 0.040).

Regarding educational level, statistically significant differences were found in the use of instrumental support (*p* = 0.030), with higher scores among parents with compulsory education (4.8; SD: 2.5) and vocational training (4.5; SD: 1.4). These parents were more likely to seek advice, assistance, and guidance from their close social network.

Parents whose child with Sturge–Weber syndrome was their firstborn displayed higher levels of denial compared to those who had the affected child in the second or third position within their family (1.4 vs. 0.1 and 0.3; *p* = 0.036). Similarly, denial was also more prevalent among parents with only one child (1.8 vs. 0.3 and 0.2).

Detailed results are provided in Supplementary Table S1.

### 3.2. Finding Meaning Through Caregiving Scale (FMTCS)

Regarding the Finding Meaning Through Caregiving Scale (FMTCS) (Table 4), the total meaning score yielded a mean of 164.50 (SD: 19.73), with values ranging from 112 to 204.

In the Provisional Meaning subscale, which assesses how participants find meaning in their daily lives through caregiving, including decision-making and recognizing positive aspects of their role, the mean score was 71.85 (SD: 7.76).

The Loss/Powerlessness subscale, which measures the exhaustion and helplessness experienced by the parents in the study, resulted in a mean score of 73.60 (SD: 14.75).

Finally, in the Ultimate Meaning subscale, which evaluates the spiritual and religious aspects that contribute to better coping with caregiving, participants scored a mean of 19.03 (SD: 7.32).

The correlations performed (Table 5) between the different subscales of the FMTCS and the ages of both parents and children suggest a statistically significant relationship between the age of the children and how parents find meaning in their daily lives through caregiving.

Specifically, significant correlations were found for both the Provisional Meaning subscale (Rs = 0.375, *p* = 0.049) and the Total Meaning score (Rs = 0.294, *p* = 0.049). These results indicate that as the affected child grows older, parents tend to experience a greater sense of meaning in their caregiving role.

#### Associations

The bivariate analysis of the Sense of Caregiving Scale (Table 6) revealed differences in the mean scores across the four subscales, although no statistically significant differences were found for any of the sociodemographic variables studied.

In the Provisional Meaning subscale, higher-than-average scores (mean: 71.85) were observed among: separated parents (77.0), Parents with primary education level (76.5 and parents with only one child (76.2)

The Loss/Powerlessness subscale, which reflects feelings of helplessness and loss of control experienced by caregivers in their decision-making process and caregiving role, showed higher scores in: nuclear families (76.5), parents working full-time (78.1) and fathers compared to mothers (78.4 vs. 71.3)

Regarding the Ultimate Meaning subscale, parents with a higher tendency to incorporate spirituality into caregiving were: separated parents (25.0) and parents whose affected child was their firstborn (23.0)

A similar trend was observed in the Total Meaning score, where:

- Parents whose affected child was their firstborn scored the highest (171.3)
- Nuclear family parents showed a higher sense of caregiving (167.6)
- Parents working full-time showed higher total meaning scores compared to other employment categories, although this difference did not reach statistical significance (*p* = 0.054).

### 3.3. Sense of Caregiving Through Coping Strategies

The correlations between the Sense of Caregiving Scale (FMTCS) and Brief Cope (Table 7) reveal significant associations like parents who exhibit a higher Provisional Meaning which reflects the ability to recognize positive aspects of their situation also demonstrate: greater active coping (Rs = 0.423; *p* = 0.025), greater acceptance (Rs = 0.562; *p* = 0.002) and higher use of humor as a coping mechanism (Rs = 0.557; *p* = 0.002).

No statistically significant associations were found between Loss/Powerlessness and any coping strategies. In the Ultimate Meaning subscale, which reflects spiritual and religious aspects of caregiving, significant correlations were identified with: positive reframing (Rs = 0.387; *p* = 0.042), religious coping (Rs = 0.785; *p* < 0.001) and self-blame (Rs = 0.572; *p* = 0.001), with the latter reflecting self-criticism and guilt experienced by parents.

Finally, the Total Meaning Score, obtained by summing the three subscales, showed a significant negative correlation with behavioral disengagement (Rs = −0.401; *p* = 0.034). This suggests that as parents develop a greater overall sense of caregiving, they are less likely to engage in behavioral disengagement, meaning they are less inclined to withdraw efforts or give up on addressing stressors.

## 4. Discussion

This study provides exploratory evidence on coping strategies and sense of caregiving among parents of children with Sturge–Weber syndrome. The findings indicate that acceptance and active coping are the most frequently used strategies, and that higher levels of caregiving meaning are associated with more adaptive coping patterns. These results suggest that meaning-making processes play a central role in parental adjustment to rare and complex pediatric conditions.

The predominance of acceptance and active coping observed in this study is consistent with previous research on caregivers of children with chronic and complex conditions, which has documented high emotional burden, anxiety, and responsibility, together with the progressive development of adaptive coping strategies over time [34,35,36]. Similarly, earlier studies in families affected by Sturge–Weber syndrome reported that acceptance tends to develop gradually as parents acquire experience and confidence in managing their child’s condition [37]. In line with these findings, the present study shows that the child’s age is associated with both coping strategies and sense of caregiving meaning, reflecting a dynamic process of psychological adaptation.

Beyond confirming previous evidence, this study contributes novel insights by jointly examining coping strategies and sense of caregiving meaning in parents of children with Sturge–Weber syndrome. While meaning-making processes have been widely explored in other chronic conditions, their relationship with coping in this specific population has received limited attention. The observed association suggests that parents who attribute positive meaning to caregiving may be better equipped to mobilize adaptive coping resources and sustain long-term engagement in care. This finding expands previous work on parental identity and expertise, in which parents progressively develop a strong sense of competence and responsibility in response to their child’s condition [36].

The diagnosis of a rare disease in a child initiates a complex emotional process characterized by fear, uncertainty, guilt, and insecurity, often intensified by limited information and unpredictable disease trajectories. These emotional reactions have been consistently described in families facing rare and chronic conditions [38,39]. In the present study, parents demonstrated an active coping style, accepting both the disease and the associated care responsibilities. This acceptance enabled them to take direct actions to manage stressors without resorting to avoidance strategies such as substance use or behavioral disengagement.

Returning home after hospitalization represents a particularly demanding transition for families, as parents become the main observers and managers of symptoms, disease progression, and potential complications, some of which may be life-threatening [35]. In this context, parental education, attitudes, and confidence play a crucial role in symptom management and decision-making. The continuous need for vigilance and responsibility may further reinforce the centrality of meaning-making processes in sustaining parental engagement over time.

In addition to medical demands, families of children with Sturge–Weber syndrome frequently encounter social and environmental barriers that affect daily functioning, social participation, and access to community resources. Difficulties in attending public spaces, benefiting from municipal services, or navigating social support systems may further intensify parental stress and contribute to feelings of isolation. Although these aspects were not directly measured in the present study, existing evidence suggests that such social constraints significantly influence parental well-being and coping processes [35]. Understanding parental adaptation within this broader social context is therefore essential for designing comprehensive support interventions.

Within this complex process, nursing professionals play a central role in supporting families. Nurses are strategically positioned to identify maladaptive coping patterns, provide emotional support, and promote adaptive strategies such as acceptance, positive reframing, and problem-solving. Previous studies have shown that structured nursing interventions can effectively reduce parenting stress and enhance family functioning [40], while high-quality communication between parents and nurses strengthens trust and facilitates shared decision-making [41]. In the context of rare diseases, where families often face uncertainty and limited professional expertise, nursing-led interventions focused on coping facilitation and meaning-making may be particularly valuable.

Sustained caregiving demands place parents at risk of emotional exhaustion and burnout, especially in the context of complex and long-term conditions. Recent conceptualizations describe parental burnout as a multidimensional phenomenon involving emotional exhaustion, role overload, and diminished personal resources [42]. Consistent with Abdoli et al. [43], many parents perceive caregiving as a non-negotiable responsibility, which may increase vulnerability to long-term psychological distress. Strengthening formal support systems, promoting shared care models, and ensuring access to psychosocial services may therefore be essential for preventing burnout and fostering sustainable caregiving.

### 4.1. Strengths and Limitations of the Study

Given the exploratory nature of this study and the small sample size, the findings should be interpreted with caution. The cross-sectional design does not allow causal relationships to be established between coping strategies and sense of caregiving meaning. In addition, recruitment through a patient association may have introduced self-selection bias, as parents with greater engagement or coping resources may have been more likely to participate. The predominance of mothers reflects persistent gender inequalities in informal caregiving roles [44,45] and may further limit the generalizability of the results. Nevertheless, despite these limitations, this study provides valuable preliminary insights into an under-researched population and contributes to the growing body of evidence on parental adaptation in rare diseases.

### 4.2. Further Research and Recommendations

Future research should prioritize longitudinal designs to examine changes in coping and meaning-making processes over time, as well as multicenter and international collaborations to overcome sample size limitations. Qualitative approaches are also needed to explore in depth the lived experiences of parents, particularly regarding social participation, access to services, and identity reconstruction. Integrating quantitative and qualitative methods may provide a more comprehensive understanding of parental adaptation. Moreover, the development and evaluation of structured nursing and psychosocial interventions represent an important research priority, with the aim of strengthening parental resources, reducing emotional burden, and promoting sustainable caregiving practices.

## 5. Conclusions

The findings of this study suggest that the sense of caregiving among parents of children with Sturge–Weber syndrome increases progressively as they adapt to their child’s condition and integrate caregiving into their daily lives. Over time, parents tend to develop greater acceptance, seek meaning in their role, and adopt adaptive coping strategies such as positive reframing, emotional expression, and humor, which contribute to emotional resilience and psychological adjustment. From a clinical perspective, these results highlight the importance of early and sustained professional support aimed at strengthening parental coping and meaning-making processes. Nursing professionals and multidisciplinary teams play a key role in identifying vulnerable families and providing tailored psychosocial interventions. These findings may inform the design of support programs focused on emotional support, education, and coping facilitation, contributing to the prevention of caregiver distress and burnout. Although the exploratory nature of this study requires cautious interpretation, the results underline the relevance of integrating emotional and practical support into routine care for families affected by rare diseases.

## Figures and Tables

**Table 1:** Description of the sample studied (global and disaggregated by parental role).

		Global (n = 28; 100%)		Mothers (n = 19; 67.9%)		Parents (n = 9; 32.1%)	
Variables		Mean (SD)	Min–Max	Mean (SD)	Min–Max	Mean (SD)	Min–Max
Age (n = 28)		43.3 (5.2)	34–54	41.84 (4.4)	34–49	46.4(5.6)	38–54
Age Child with SWS		8 (5.2)	1–21	7.3 (4.6)	1–18	9.44 (6.2)	3–21
**Variable**	**Categories**	**n**	**%**	**n**	**%**	**n**	**%**
Family type	Nuclear	23	82.1%	14	73.7%	9	100%
	Single-parent	3	10.7%	3	15.8%		
	Separated parents	2	7.1%	2	10.5%		
Employment status	CUME Subsidy	3	10.7%	3	15.8%		
	Unemployment	5	17.9%	4	21.1%	1	11.1%
	Part-time employment	3	10.7%	3	15.8%		
	Full-time employment	17	60.7%	9	47.4%	8	88.9%
Educational level	Primary	4	14.3%	3	15.8%	1	11.1%
	Secondary	5	17.9%	4	21.1%	1	11.1%
	Vocational training	6	21.4%	5	26.3%	1	11.1%
	University	13	46.4%	7	36.8%	6	66.7%
Number of children	1 Child	4	14.3%	3	15.8%	1	11.1%
	2 children	15	53.6%	8	42.1%	7	77.8%
	3 children	9	32.1%	8	42.1%	1	11.1%
Birth order of child with SWS	First child	7	25%	4	21.1%	3	33.3%
	Second child	14	50%	9	47.4%	5	55.6%
	Third child	7	25%	6	31.6%	1	11.1%

SD (standard deviation).

**Table 2:** Description of Brief COPE.

Coping Styles	Mean	SD	Median	Minimum	Maximum	IQR
Active Coping	5.07	1.120	5.50	2	6	2
Planning	3.79	1.813	4.00	0	6	3
Use of instrumental support	3.75	1.713	4.00	1	6	3
Use of emotional support	3.89	1.474	4.00	2	6	2
Self-distraction	2.18	1.657	2.00	0	6	2
Venting	2.46	1.774	2.00	0	6	3
Behavioral disengagement	0.61	1.166	0.00	0	4	1
Positive reframing	4.07	1.676	4.00	0	6	2
Denial	0.50	1.036	0.00	0	4	1
Acceptance	5.14	0.848	5.00	3	6	1
Religion	1.57	2.168	0.00	0	6	4
Substance abuse	0.04	0.189	0.00	0	1	0
Humor	2.18	1.982	2.00	0	6	4
Self-blame	1.61	1.449	1.00	0	5	2

SD (standard deviation), Interquartile Range (IQR).

**Table 3:** Age and Age Child with SWS correlation with Brief Cope.

		Age	Age Child with SWS
Variable	Categories		
Active coping	Spearman correlation	0.098	0.335
	*p* Value	0.618	0.081
Planning	Spearman correlation	0.309	0.342
	*p* Value	0.109	0.075
Use of instrumental support	Spearman correlation	0.351	0.512
	*p* Value	0.067	0.005
Use of emotional support	Spearman correlation	0.160	0.441
	*p* Value	0.417	0.019
Self-distraction	Spearman correlation	0.258	0.260
	*p* Value	0.184	0.182
Venting	Spearman correlation	0.518	0.542
	*p* Value	0.005	0.003
Behavioral disengagement	Spearman correlation	0.177	−0.161
	*p* Value	0.367	0.414
Positive reframing	Spearman correlation	0.324	0.397
	*p* Value	0.093	0.036
Denial	Spearman correlation	−0.084	−0.062
	*p* Value	0.670	0.752
Acceptance	Spearman correlation	0.024	0.397
	*p* Value	0.903	0.036
Religion	Spearman correlation	0.096	0.232
	*p* Value	0.627	0.236
Substance abuse	Spearman correlation	0.095	0.132
	*p* Value	0.629	0.504
Humor	Spearman correlation	0.449	0.501
	*p* Value	0.016	0.007
Self-blame	Spearman correlation	0.334	0.277
	*p* Value	0.082	0.154

**Table 4:** Description of FMTCS.

Subscales	Mean	SD	Median	Minimum	Maximum	IQR
Provisional meaning	71.85	7.76	74.50	51	85	9
Loss/Powerlessness	73.60	14.75	74.00	43	95	22.50
Ultimate meaning	19.03	7.32	18.50	10	35	12.75
Total meaning	164.50	19.73	163.00	112	204	28

SD (standard deviation), Interquartile Range (IQR).

**Table 5:** Age and Age Child with SWS correlation with FMTCS.

Variable	Categories	Age	Age Child with SWS
Provisional Meaning	Spearman correlation	0.105	0.375
	*p* Value	0.594	0.049
Loss/ Powerlessness	Spearman correlation	−0.046	0.083
	*p* Value	0.815	0.673
Ultimate Meaning	Spearman correlation	0.163	0.271
	*p* Value	0.407	0.163
Total Meaning	Spearman correlation	0.067	0.294
	*p* Value	0.734	0.049

**Table 6:** Bivariate analysis FMCTS.

			Provisional Meaning		Loss/Powerlessness		Ultimate Meaning		Total Meaning	
Variable	Categories	n	Mean (SD)	*p* Value	Mean (SD)	*p* Value	Mean (SD)	*p* Value	Mean (SD)	*p* Value
Parental role	Mother	19	72.0 (8.9)	0.383	71.3 (14.3)	0.223	19.4 (7.3)	0.772	162.7 (20.4)	0.595
	Father	9	71.6 (5.2)		78.4 (15.3)		18.3 (7.7)		168.3 (18.9)	
Family type	Nuclear	23	72.2 (7.2)	0.220	76.5 (12.6)	0.138	18.9 (7.4)	0.546	167.6 (17.9)	0.442
	Single-parent	3	66.0 (13.1)		63.7 (21.2)		16.3 (4.5)		146.0 (32.2)	
	Separated parents	2	77.0 (1.4)		55.0 (16.8)		25.0 (9.9)		157.0 (8.5)	
Employment status	CUME Subsidy	3	66.7 (9.1)	0.520	65.0 (8.0)	0.256	18.3 (6.0)	0.402	150.0 (8.5)	0.054
	Unemployment	5	71.4 (7.8)		71.2 (12.9)		14.8 (4.9)		157.4 (11.2)	
	Part-time employment	3	68.0 (14.7)		60.7 (25.6)		18.7 (11.5)		147.3 (33.7)	
	Full-time employment	17	73.6 (6.2)		78.1 (13.0)		20.5 (7.5)		172.2 (17.4)	
Educational level	Primary	4	76.5 (2.5)	0.564	63.5 (16.5)	0.582	22.2 (11.3)	0.848	162.3 (20.9)	0.708
	Secondary	5	74.4 (2.5)		78.6 (12.9)		18.0 (6.1)		171.0 (8.0)	
	Vocational training	6	70.0 (12.8)		74.0 (18.6)		17.7 (7.6)		161.7 (32.1)	
	University	13	70.3 (7.0)		74.6 (13.2)		19.1 (6.9)		164.0 (17.1)	
Number of children	1 Child	4	76.2 (0.9)	0.212	70.5 (25.9)	0.968	19.0 (9.8)	0.863	165.8 (17.7)	0.594
	2 children	15	72.5 (8.2)		74.5 (13.8)		19.7 (7.6)		166.7 (23.3)	
	3 children	9	68.8 (8)		73.6 (11.9)		17.9 (6.4)		160.2 (14.7)	
Birth order of child with SWS	First child	7	74.7 (3.1)	0.519	73.6 (22.5)	0.911	23.0 (9.1)	0.344	171.3 (22.6)	0.697
	Second child	14	71.6 (9.3)		73.1 (11.7)		17.1 (6.3)		161.9 (20.8)	
	Third child	7	69.4 (7.7)		74.6 (13.3)		18.9 (6.6)		162.9 (15.2)	

SD (standard deviation).

**Table 7:** Correlation of FMTCS with Brief Cope.

		Provisional Meaning	Loss/ Powerlessness	Ultimate Meaning	Total Meaning
Variable	Categoría				
Active coping	Correlación Spearman	0.423	−0.130	−0.177	−0.016
	Valor P	0.025	0.509	0.367	0.936
Planning	Correlación Spearman	0.361	−0.128	0.363	0.159
	Valor P	0.059	0.517	0.058	0.419
Use of instrumental support	Correlación Spearman	0.175	−0.212	0.359	0.026
	Valor P	0.373	0.279	0.061	0.896
Use of emotional support	Correlación Spearman	0.260	−0.084	0.356	0.173
	Valor P	0.182	0.671	0.063	0.378
Self-distraction	Correlación Spearman	0.057	−0.221	0.087	−0.163
	Valor P	0.772	0.259	0.660	0.408
Venting	Correlación Spearman	0.323	0.237	0.259	−0.050
	Valor P	0.094	0.226	0.182	0.800
Behavioral disengagement	Correlación Spearman	−0.142	−0.362	−0.111	−0.401
	Valor P	0.470	0.058	0.574	0.034
Positive Reframing	Correlación Spearman	0.329	0.037	0.387	0.328
	Valor P	0.087	0.851	0.042	0.088
Denial	Correlación Spearman	0.173	−0.337	0.122	−0.311
	Valor P	0.380	0.080	0.536	0.108
Acceptance	Correlación Spearman	0.562	0.190	−0.041	0.293
	Valor P	0.002	0.333	0.836	0.130
Religion	Correlación Spearman	0.087	−0.183	0.785	0.209
	Valor P	0.659	0.352	0.000	0.286
Substance abuse	Correlación Spearman	0.096	−0.322	0.287	−0.155
	Valor P	0.628	0.095	0.139	0.431
Humor	Correlación Spearman	0.557	0.091	0.151	0.271
	Valor P	0.002	0.645	0.443	0.163
Self-blame	Correlación Spearman	0.179	0.006	0.572	0.245
	Valor P	0.363	0.977	0.001	0.208

## Data Availability

The research data associated with this study are available for consultation in the Zenodo repository (EU Open Research Repository). https://doi.org/10.5281/zenodo.14836523 (accessed on 12 Februay 2026).

## Supplementary Materials

The following supporting information can be downloaded at: https://www.mdpi.com/article/10.3390/nursrep16020066/s1, Table S1: Bivariate analysis Brief COPE.

## Public Involvement Statement

No public involvement in any aspect of this research.

## Guidelines and Standards Statement

This manuscript was drafted in accordance with the STROBE Statement (Strengthening the Reporting of Observational Studies in Epidemiology) for cross-sectional observational studies.

## Abbreviations

The following abbreviations are used in this manuscript:

SWS	Sturge–Weber Syndrome
FMTCS	Finding Meaning Through Caregiving Scale
AESSW	Asociación Española de Síndrome de Sturge–Weber
SD	Standard Deviation
IQR	Interquartile Range
