# Supplementary material for: Coping Strategies and Sense of Care Among Parents of Children Affected by Sturge–Weber Syndrome: A Cross-Sectional Study
Source: Nurs Rep. 2026 Feb 14;16(2):66. doi: 10.3390/nursrep16020066 (PMC12943280; doi:10.3390/nursrep16020066)
Supplement: Supplementary file 1 [file nursrep-16-00066-s001.zip › nursrep-4127125-supplementary.pdf]

Supplementary Table S1. Bivariate analysis Brief COPE.

			Active coping		Planning		Use of instrumental support		Use of emotional support		Self-distraction	
**Variable**	**Categories**	**n**	**Mean (SD)**	**P Value**	**Mean (SD)**	**P Value**	**Mean (SD)**	**P Value**	**Mean (SD)**	**P Value**	**Mean (SD)**	**P Value**
Parental role	Mother	19	5.3 (1.0)	0.263	3.8 (1.9)	0.735	3.7 (1.9)	0.962	3.9 (1.4)	0.962	2.5 (1.7)	0.188
	Father	9	4.7 (1.3)		3.7 (1.6)		3.8 (1.6)		3.9 (1.7)		1.6 (1.3)	
Family type	Nuclear	23	4.9 (1.2)	0.229	3.7 (1.9)	0.235	3.7 (1.7)	0.819	3.9 (1.5)	0.781	1.8 (1.5)	0.035
	Single-parent	3	5.7 (0.6)		3.0 (1.0)		4.3 (1.2)		3.7 (1.2)		3.0 (1.0)	
	Separated parents	2	6.0 (0.0)		5.5 (0.7)		4.0 (2.8)		4.5 (0.7)		5.0 (0.0)	
Employment status	CUME Subsidy	3	5.0 (1.0)	0.091	3.7 (2.1)	0.505	4.0 (1.7)	0.298	3.3 (1.5)	0.648	1.7 (0.6)	0.226
	Unemployment	5	4.8 (1.3)		2.4 (2.2)		2.4 (1.7)		3.6 (1.1)		1.2 (1.3)	
	Part time employment	3	6.0 (0.0)		4.0 (1.7)		4.0 (2.6)		3.3 (1.5)		3.7 (1.5)	

	Full time employment	17	5.0 (1.1)		4.2 (1.6)		4.1 (1.5)		4.2 (1.6)		2.3 (1.8)	
Educational level	Primary	4	6.0 (0.0)	0.184	3.8 (2.9)	0.424	4.8 (2.5)	0.030	5.3 (1.0)	0.059	2.8 (3.2)	0.339
	Secondary	5	4.8 (0.8)		2.6 (2.2)		1.8 (0.8)		3.0 (1.7)		1.0 (1.0)	
	Vocational training	6	5.2 (1.3)		4.3 (1.0)		4.5 (1.4)		4.5 (1.0)		2.2 (1.3)	
	University	13	4.9 (1.2)		4.0 (1.6)		3.9 (1.3)		3.5 (1.4)		2.5 (1.3)	
Number of children	1 offspring	4	6.0 (0.0)	0.070	4.5 (1.3)	0.533	4.0 (2.2)	0.651	4.0 (1.4)	0.361	3.0 (1.4)	
	2 offsprings	15	5.1 (1.2)		3.4 (2.0)		3.9 (1.8)		4.2 (1.5)		2.0 (2.0)	0.431
	3 offsprings	9	4.7 (1.0)		4.1 (1.0)		3.3 (1.4)		3.3 (1.4)		2.1 (0.9)	
Birthorder of offspring with SWS	First child	7	5.3 (0.9)	0.328	4.4 (1.3)	0.580	4.1 (1.6)	0.502	4.3 (1.4)	0.154	2.7 (1.4)	0.344
	Second child	14	5.1 (1.4)		3.4 (2.1)		3.9 (2.0)		4.1 (1.5)		1.9 (2.0)	
	Third child	7	4.7 (0.8)		3.9 (1.7)		3.1 (1.0)		3.0 (1.4)		2.3 (1.0)	

SD (standard deviation)

Table S1: Bivariate analysis Brief Cope. Continuation

			Venting		Behavioral disengagement		Positive reframing		Denial		Acceptance	
**Variable**	**Categories**	**n**	**Mean (SD)**	**p Value**	**Mean (SD)**	**p Value**	**Mean (SD)**	**p Value**	**Mean (SD)**	**P Value**	**Mean (SD)**	**p Value**
Parental role	Mother	19	2.6 (1.8)	0.629	0.5 (1.1)	0.562	4.0 (1.7)	0.562	0.7 (1.2)	0.357	5.3 (1.1)	0.263
	Father	9	2.2 (1.8)		0.9 (1.8)		4.2 (1.7)		0.1 (0.3)		4.8 (0.7)	
Family type	Nuclear	23	2.3 (1.6)	0.085	0.5 (1.0)	0.132	4.2 (1.5)	0.635	0.2 (0.5)	0.055	5.1 (0.9)	0.791
	Single-parent	3	2.0 (1.7)		2.0 (2.0)		3.7 (1.2)		2.0 (2.0)		5.0 (1.0)	
	Separated parents	2	5.5 (0.7)		0.0 (0.0)		3.0 (4.2)		1.5 (2.1)		5.5 (0.7)	
Employment status	CUME Subsidy	3	1.7 (0.6)	0.414	0.0 (0.0)	0.667	3.7 (0.6)	0.005	0.3 (0.6)	0.251	5.0 (1.0)	0.900

	Unemployment	5	1.6 (2.2)		1.0 (1.7)		2.6 (2.1)		0.8 (1.8)		5.4 (0.6)	
	Part time employment	3	3.0 (2.6)		0.7 (1.2)		2.0 (3.5)		1.7 (1.5)		5.0 (1.0)	
	Full time employment	17	2.8 (1.6)		0.6 (1.1)		4.9 (1.0)		0.2 (0.6)		5.1 (0.9)	
Educational level	Primary	4	4.0 (2.7)	0.125	0.8 (1.5)	0.895	3.5 (2.6)	0.612	0.8 (1.5)	0.218	5.3 (0.5)	0.941
	Secondary	5	1.2 (0.8)		0.2 (0.4)		3.6 (1.9)		0.0 (0.0)		5.2 (0.4)	
	Vocational training	6	3.2 (1.5)		1.0 (1.7)		4.8 (1.2)		1.3 (1.6)		5.3 (0.8)	
	University	13	2.2 (1.5)		0.5 (1.1)		4.1 (1.5)		0.2 (0.4)		5.0 (1.1)	
Number of children	1 offspring	4	3.5 (1.9)	0.342	1.0 (2.0)	0.959	3.3 (2.4)	0.658	1.8 (2.1)	0.260	5.8 (0.5)	0.178
	2 offsprings	15	2.5 (2.0)		0.5 (1.0)		4.2 (1.8)		0.3 (0.7)		5.1 (0.8)	
	3 offsprings	9	1.9 (1.1)		0.6 (1.1)		4.2 (1.1)		0.2 (0.4)		4.9 (0.9)	
	First child	7	3.0 (1.6)	0.405	0.9 (1.6)	0.846	4.0 (2.0)	0.991	1.4 (1.6)	0.036	5.6 (0.5)	0.156

Birthorder of offspring with SWS	Second child	14	2.6 (2.1)	0.4 (0.9)	4.0 (1.8)	0.1 (0.5)	5.1 (0.9)
	Third child	7	1.7 (0.8)	0.7 (1.3)	4.3 (1.3)	0.3 (0.5)	4.7 (1.0)

SD (standard deviation)

Table S1: Bivariate analysis Brief Cope. Continuation

			Religion		Substance abuse		Humor		Self-blame	
**Variable**	**Categories**	**n**	**Mean (SD)**	**p Value**	**Mean (SD)**	**p Value**	**Mean (SD)**	**p Value**	**Mean (SD)**	**p Value**
Parental role	Mother	19	1.6 (2.2)	1.000	0.1 (0.2)	0.847	2.0 (2)	0.357	1.8 (1.6)	0.285
	Father	9	1.6 (2.2)		0.0 (0.0)		2.7 (2)		1.1 (1.1)	
Family type	Nuclear	23	1.6 (2.1)	0.100	0.0 (0.0)	0.002	2.3 (2.0)	0.707	1.5 (1.4)	0.275

	Single-parent	3	0.0 (0.0)		0.0 (0.0)		1.3 (2.3)		1.3 (0.6)	
	Separated parents	2	4.0 (2.8)		0.5 (0.7)		2.0 (2.8)		3.5 (2.1)	
Employment status	CUME Subsidy	3	1.7 (2.1)		0.0 (0.0)		1.7 (1.5)		1.3 (1.5)	
	Unemployment	5	0.0 (0.0)	0.251	0.0 (0.0)	0.040	1.4 (1.9)	0.231	0.6 (0.9)	0.142
	Part time employment	3	2.0 (3.4)		0.3 (0.6)		0.7 (1.2)		2.0 (2.6)	
	Full time employment	17	1.9 (2.2)		0.0 (0.0)		2.8 (2.0)		1.9 (1.3)	
Educational level	Primary	4	2.5 (3.0)		0.3 (0.5)		3.0 (2.2)		2.3 (2.6)	
	Secondary	5	1.0 (1.7)	0.626	0.0 (0.0)	0.112	1.2 (1.8)	0.555	0.8 (0.4)	0.537
	Vocational training	6	1.0 (2.4)		0.0 (0.0)		2.2 (2.6)		1.7 (1.9)	
	University	13	1.8 (2.0)		0.0 (0.0)		2.3 (1.8)		1.7 (1.0)	
Number of children	1 offspring	4	1.5 (3.0)	0.885	0.3 (0.5)	0.050	2.3 (1.7)	0.418	1.8 (2.4)	0.958

	2 offsprings	15	1.7 (2.3)		0.0 (0.0)		2.6 (2.2)		1.6 (1.4)	
	3 offsprings	9	1.3 (1.8)		0.0 (0.0)		1.4 (1.7)		1.4 (1.1)	
Birthorder of offspring with SWS	First child	7	3.1 (3.0)	0.204	0.1 (0.4)	0.223	2.3 (1.9)	0.541	2.3 (2.1)	0.425
	Second child	14	0.8 (1.3)		0.0 (0.0)		2.5 (2.1)		1.2 (1.1)	
	Third child	5	1.6 (2.2)		0.0 (0.0)		1.4 (1.8)		1.7 (1.1)	

SD (standard deviation)
